# ULK1 methylation promotes TGF‐β1‐induced endometrial fibrosis via the FOXP1/DNMT1 axis

**DOI:** 10.1002/kjm2.12915

**Published:** 2024-12-04

**Authors:** Yuhua Zeng, Qing Feng

**Affiliations:** ^1^ Health Management Medicine Center The Third Xiangya Hospital of Central South University Changsha China; ^2^ Department of Obstetrics and Gynecology The Third Xiangya Hospital of Central South University Changsha China

**Keywords:** autophagy, FOXP1, IUA, methylation, ULK1

## Abstract

Intrauterine adhesion (IUA) is the second most common cause of secondary infertility in women and can also lead to menstrual abnormalities and multiple adverse pregnancy outcomes. Therefore, elucidating the mechanism of its development is crucial for the prevention and treatment of IUA. This study will investigate the function and mechanism of forkhead box P1 (FOXP1)/DNA methyltransferase 1 (DNMT1)/unc‐51‐like autophagy activating kinase 1 (ULK1) in IUA. Expression levels of key genes were detected using western blot and quantitative ‐ real time reverse transcription polymerase chain reaction. Cell proliferation was detected by CCK‐8 and EdU staining. Transcriptional regulation relationships were detected by dual luciferase reporter gene and chromatin immunoprecipitation (ChIP) assay. Methylation station of ULK1 was detected by methylmion specific PCR (MSP). Fibrosis and pathological changes in the uterine cavity tissues were detected by Masson and hematoxylin and eosin staining. It was observed that the expression of FOXP1 and DNMT1 increased in transforming growth factor (TGF)‐β1‐induced cells, while ULK1 expression decreased. Downregulation of FOXP1 could inhibit human endometrial stromal cells proliferation and autophagy, as well as decrease the expression of fibrogenic factors (collagen type I alpha 1 chain [COL1A1], fibronectin [FN], and alpha‐smooth muscle actin [α‐SMA]). The results of MSP and ChIP experiments showed that DNMT1 promotes methylation of the ULK1 promoter region and inhibits its transcription. In an animal model, knockdown of FOXP1 alleviated pathological fibrosis and uterine adhesions. Knockdown of FOXP1 can inhibit endometrial fibrosis in IUA rats; FOXP1 could be a potential target for the treatment of IUA.

**Abbreviation**
ChIP: chromatin immunoprecipitation
DNMT: DNA methyltransferase
ECM: extracellular matrix
FOXP1: forkhead box P1
IUA: intrauterine adhesion
MSP: methylmion specific PCR
ULK1: unc‐51‐like autophagy activating kinase 1

## 1 INTRODUCTION

Intrauterine adhesion (IUA) is a gynecologic condition in which the anterior and posterior walls of the uterine cavity are partially or completely adherent due to the damage of the basal layer of the endometrium.^1^ In recent years, fibroblast activation in the development of IUA has received widespread attention and become a hot spot for research.^2^ Additionally, emerging evidence suggests that autophagic dysfunction is closely associated with excessive extracellular matrix (ECM) deposition and fibrosis, resulting in a significant increase in the expression of ECM.^3^, ^4^, ^5^ However, our understanding of the precise role and underlying mechanisms of autophagy in endometrial fibrosis remains limited.

Unc‐51‐like autophagy activating kinase 1 (ULK1) is a cytoplasmic kinase that forms a complex with mammalian ATG1 protein and plays a crucial role in the initiation of autophagy.^6^ Despite its importance, the precise role of ULK1 has remained elusive, although some studies have shed light on its involvement in fibrotic diseases. For instance, research has shown that miRNA‐1297 can inhibit cardiac fibrosis by targeting ULK1.^7^ Similarly, reduced expression of ULK1 has been observed in renal biopsy tissues of diabetic patients, resulting in autophagy defects. Restoring ULK1 expression has been found to significantly alleviate diabetic nephropathy.^8^ DNA methylation, catalyzed by DNA methyltransferase (DNMT), normally represses the transcription of target genes and downregulates their expression.^9^ In the ULK1 promoter region, computational predictions suggest the presence of a cytosine phosphodiester bond guanine (CpG) island. However, it remains unknown what role DNA methylation‐modified ULK1 plays in the context of IUA.

Forkhead box P1 (FOXP1) is a transcription factor with multiple functions.^10^, ^11^ A recent study found that FOXP1 expression levels were substantially increased in endometrial mesenchymal cells of patients with endometriosis.^12^ In addition, FOXP1 promotes endometriosis fibrosis through activation of the wingless related integration site/β‐catenin signaling pathway.^13^ It is also reported that FOXP1 could inhibit autophagy.^14^, ^15^ However, no correlation between FOXP1 and IUA has been reported. According to the prediction, FOXP1 has a binding relationship with DNMT1.

Further studies suggest that FOXP1 can target and promote the expression of DNMT1, which can methylation modify and inhibit the transcription of ULK1, inhibit autophagy, and ultimately promote the disease progression of IUA. FOXP1 is expected to be a potential therapeutic target for clinical overcoming of IUA.

## 2 MATERIALS AND METHODS

### 2.1 Human endometrial stromal cell culture cell transfection

Human endometrial stromal cells (hESC) were purchased from American Type Culture Collection (ATCC, Virginia, USA). The culture medium contains 10% fetal bovine serum (minimum essential medium + non‐essential amino acids + 1% penicillin–streptomycin), and the cell culture conditions were 5% CO_2_ and a constant temperature incubator at 37°C. The IUA cell model was constructed using gradient concentrations of TGF‐β1 (5 ng/mL, 10 ng/mL, 20 ng/mL, #100‐21, Pepro Tech) for 24 h.

Overexpressed DNMT1 was synthesized by Sangon Biotech (Shanghai, China). Specific short hairpin RNAs (shRNAs) against FOXP1 (sh‐FOXP1), DNMT1 (sh‐DNMT1), and controlled shRNA (sh‐negative control) were purchased from Ribobio (Guangzhou, China). Transfection was performed utilizing Lipofectamine™ 2000 (Invitrogen) following the manufacturer's protocol for 48 h.

### 2.2 IUA rat model

The female virgin Sprague Dawley (SD) rats, with the weight around 250–280 g, were purchased from Hunan Silaikejingda Experimental Animal Co., Ltd. and kept in a specific pathogen free environment. The rats were randomly divided into model groups (five rats in each group), and the IUA model was prepared by referring to the literature,^16^ of which the medical sterile surgical wires were soaked in lipopolysaccharide (6 mg/L) for 24 h. The rat was anesthetized with 1% sodium pentobarbital (50 mg/kg), an incision was made in the middle part of the lower abdomen, and a 4 mm incision was made about 5 mm above the cervix after finding the rat's V‐shaped uterus. A scraping spoon was used to scrape the uterus through the incision, and the scraping was stopped when there was a rough feeling on the surface of the uterine cavity. The model was successfully constructed when the uterine cavity was dissected to show the disappearance of tubular structures.

### 2.3 Cell counting kit (CCK)‐8 assay

The cells were counted and seeded on a 96‐well plate with a total amount of 5000 cells. Before cell viability assay at each time point, 10 μL of CCK‐8 solution (C0037, Beyotime) was added to each well, and the absorbance at 450 nm was measured by a microplate reader (ELx800, BioTek) after 2 h of continued incubation.

### 2.4 Flow cytometry

The cell apoptosis in each treatment group was detected using the Annexin V‐FITC/PI apoptosis kit (AT107, Multi Sciences, China). All operations were carried out in strict accordance with the instructions.

### 2.5 Masson staining

Paraffin sections were dewaxed and rehydrated, then washed with phosphate buffered saline and stained with Weigert's iron hematoxylin staining solution for 10 min. The sections were incubated in several solutions, including an acidic alcohol solution for 5–15 s, Masson bluing solution for 3 min, Lichon red magenta staining solution for 5–10 min, weak acid working solution for 1 min, phosphomolybdic acid solution for 1 min, and aniline blue staining solution for 1–2 min. Finally, the sections were rinsed with a weak acid working solution for 1 min. The films were sealed with neutral resin.

### 2.6 Western blot

Cells were lysed using radio immunoprecipitation assay buffer lysis solution (P0013B, Beyotime, Shanghai, China) to obtain protein samples. After measuring the protein concentration using the BCA kit (P0009, Beyotime), the corresponding volume of protein was taken and mixed with the loading buffer (P0015, Beyotime), and heated in a boiling water bath for 5 min to denature the protein. The membrane transfer was performed in an ice bath with a transfer current of 220 mA and a time of 120 min. After the transfer, the membrane was rinsed in washing solution for 1–2 min, and then the membrane was placed in 5% bovine serum albumin for 60 min at room temperature. After being incubated with primary antibody on the shaker overnight at 4°C, the membrane was transferred into the secondary antibody. The membranes were photographed by chemiluminescence imaging system (Bio‐rad). The antibodies used for western blot in this study are shown in Table S1.

### 2.7 Quantitative real‐time polymerase chain reaction (PCR)

TRIZOL reagent (Invitrogen) was adopted to extract total RNA from tissues or cells. Reverse transcription was performed using a cDNA synthesis kit (Toyobo, Osaka, Japan). DNA was quantified by Fast SYBR Green Master Mix (Applied Biosystems, Foster City, CA, USA) under LC480 Real‐Time PCR Detection System (Roche, Basel, Switzerland). Primer sequences are shown in Table S2. Glyceraldehyde‐3‐phosphate dehydrogenase was utilized as an internal reference.

### 2.8 Immunofluorescence staining

Uterine tissue and cell suspensions (1 × 10^5^/mL) seeded on 24‐well plates were used as the subjects for the experimental study. Briefly, the cells were fixed using a 4% paraformaldehyde solution for 30 min and then incubated with 0.1% TritonX‐100 for 20 min. After being treated with 10% goat serum for 30 min, primary antibodies diluted with 10% goat serum were added and incubated overnight at 4°C. The fluorescent secondary antibody diluted in 10% goat serum was added and incubated at 37°C for 1 h, which was followed with 4',6‐diamidino‐2‐phenylindole staining. The stained cells were observed and photographed using a fluorescence microscope.

### 2.9 EdU staining

EdU solution (ST067, Beyotime) was added to the 24‐well plate in a ratio of 1:2000. After the cells were labeled with EdU, the culture medium was discarded, and 1 mL of 4% paraformaldehyde was added and incubated at room temperature for 15 min. The cells were then washed three times with 1 mL of PBS for 3–5 min per well. The nuclei were stained with 1000× Hoechst 33342 (#62249, ThermoFisher, USA) at room temperature for 10 min. The cells were observed and photographed using a fluorescence microscope.

### 2.10 Hematoxylin and eosin staining

Paraffin sections were stained with hematoxylin and eosin (H&E),^17^ and then sealed with neutral resin.

### 2.11 Chromatin immunoprecipitation technique (experiment)

Follow the chromatin immunoprecipitation technique (ChIP) kit (#56383, CST) instructions; 1% formaldehyde was used to bind the proteins to DNA. Terminate the cross‐link with glycine and then lyse the cells under sonication to shear the chromatin. Next, reserve a 2% input control sample and bind the DNA‐containing chromatin with FOXP1 antibody, IgG antibody, and Protein G magnetic beads to precipitate it down. Purify the DNA by removing the bound proteins through decrosslinking. Finally, use the obtained DNA as a template for RT‐PCR amplification of multiple estrogen responsive element on the DNMT1 promoter.

### 2.12 Methylmion‐specific PCR detects methylation levels

Genomic DNA was extracted following the instructions of the DNA extraction kit (DP304, TianGen, Beijing, China). The purity and concentration of DNA were detected using a UV spectrophotometer, and then genomic DNA was modified by heavy sulfite according to the instructions of the methylation kit (ME10025, ThermoFisher). The amplified products were electrophoresed in a 2% agarose gel containing ethidium bromide, and photographs were taken using a gel imaging system. The level of methylation at each ULK1 site was automatically analyzed using PyroMark Q48‐CpG software.

### 2.13 Dual luciferase reporter gene

The binding sites of FOXP1 to the DNMT1 promoter region were predicted using the online database JASPAR (https://jaspar.genereg.net/analysis). Based on the predicted results, wild and mutant sequences of the binding site (psicheck2‐DNMT1‐WT and psicheck2‐DNMT1‐MUT) were designed and synthesized, respectively. The wild and mutant sequences were then inserted into the luciferase reporter gene vector and co‐transfected with Vector and FOXP1, respectively, in human embryonic kidney 293T cells. Renilla luciferase activity was used as an internal reference, and the ratio of Firefly luciferase to Renilla luciferase activity was used as the relative luciferase activity. The experiment was conducted with three replicates.

### 2.14 Data analysis

The data were expressed as mean ± standard deviation, and one‐way analysis of variance was performed using Graphpad Prism 8.0 software. The correlation results were then analyzed using the Turkey method for multiple comparisons of the means of each group. A *p* value less than 0.05 indicated statistical significance.

## 3 RESULTS

### 3.1 TGF‐β1 was utilized to construct IUA cell model

**FIGURE 1:**
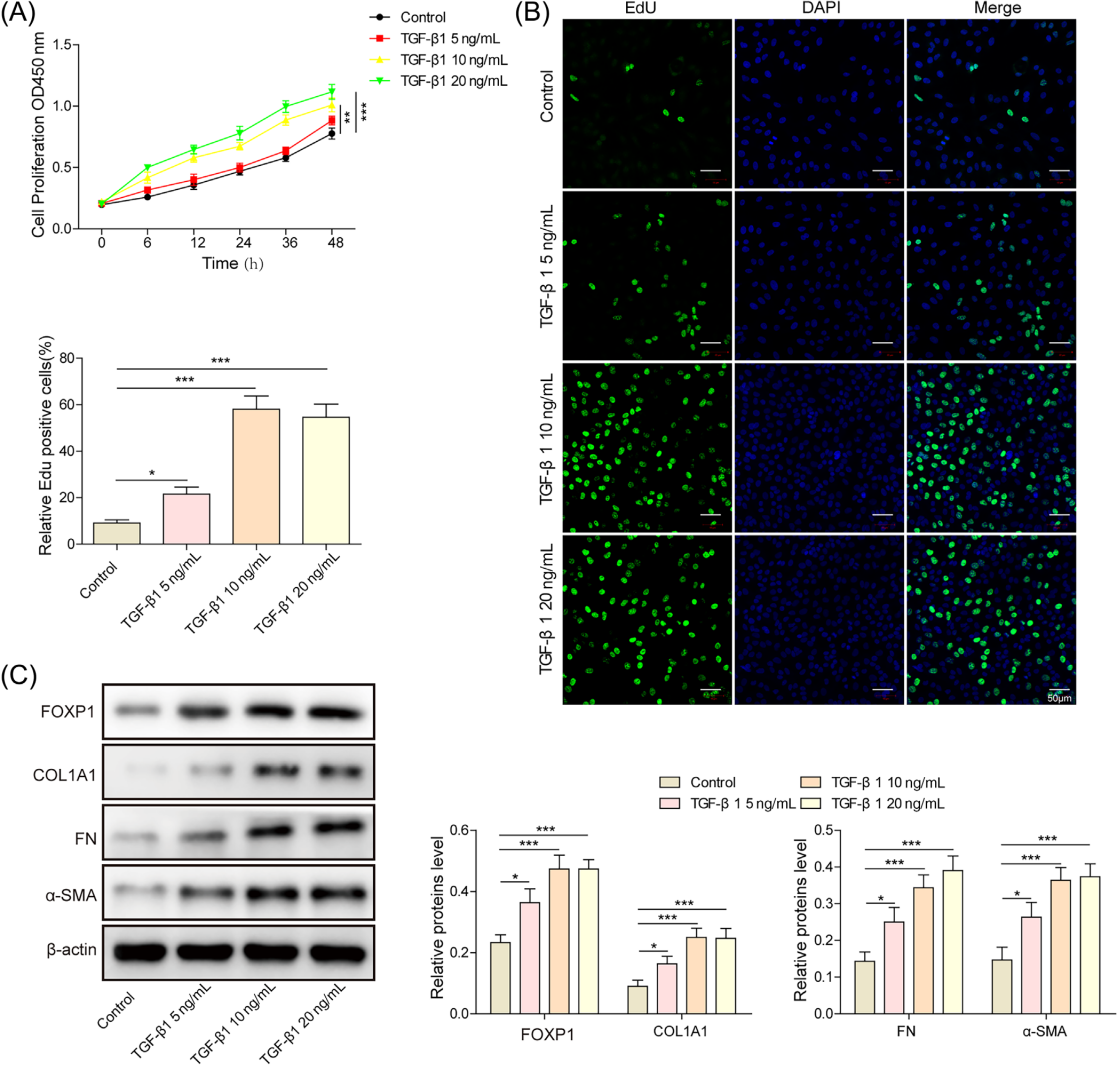
TGF‐β1 was utilized to construct intrauterine adhesion cell model. (A) CCK‐8 for cell viability; (B) EdU staining for cell proliferation ability; (C) Western blot for expression of forkhead box P1 (FOXP1), collagen type I alpha 1 chain (COL1A1), FN, and α‐SMA. *p < 0.05, **p < 0.01, and ***p < 0.001.

Since IUA is essentially an endometrial fibrosis, a series of concentrations of TGF‐β1 was added into the culture medium to construct a fibrosis cell model and explore the optimal concentration. Following TGF‐β1 treatment, cell proliferation viability increased significantly (Figure 1A,B), and the expression of FOXP1 as well as fibrosis factor increased (Figure 1C) in a concentration‐dependent manner. Once the TGF‐β1 concentration reached 10 ng/mL, the trend of increasing cell proliferation viability and related protein expression was no longer significant. Thus, this concentration was chosen for subsequent construction of the IUA model. Taken together, the optimal concentration of TGF‐β1 to induce cell inflammation was 10 ng/mL, which could upregulate the expression of FOXP1 and fibrosis factors.

### 3.2 FOXP1 inhibits autophagy and promotes fibrosis in TGF‐β1‐treated hESC through transcriptionally upregulating DNMT1

**FIGURE 2:**
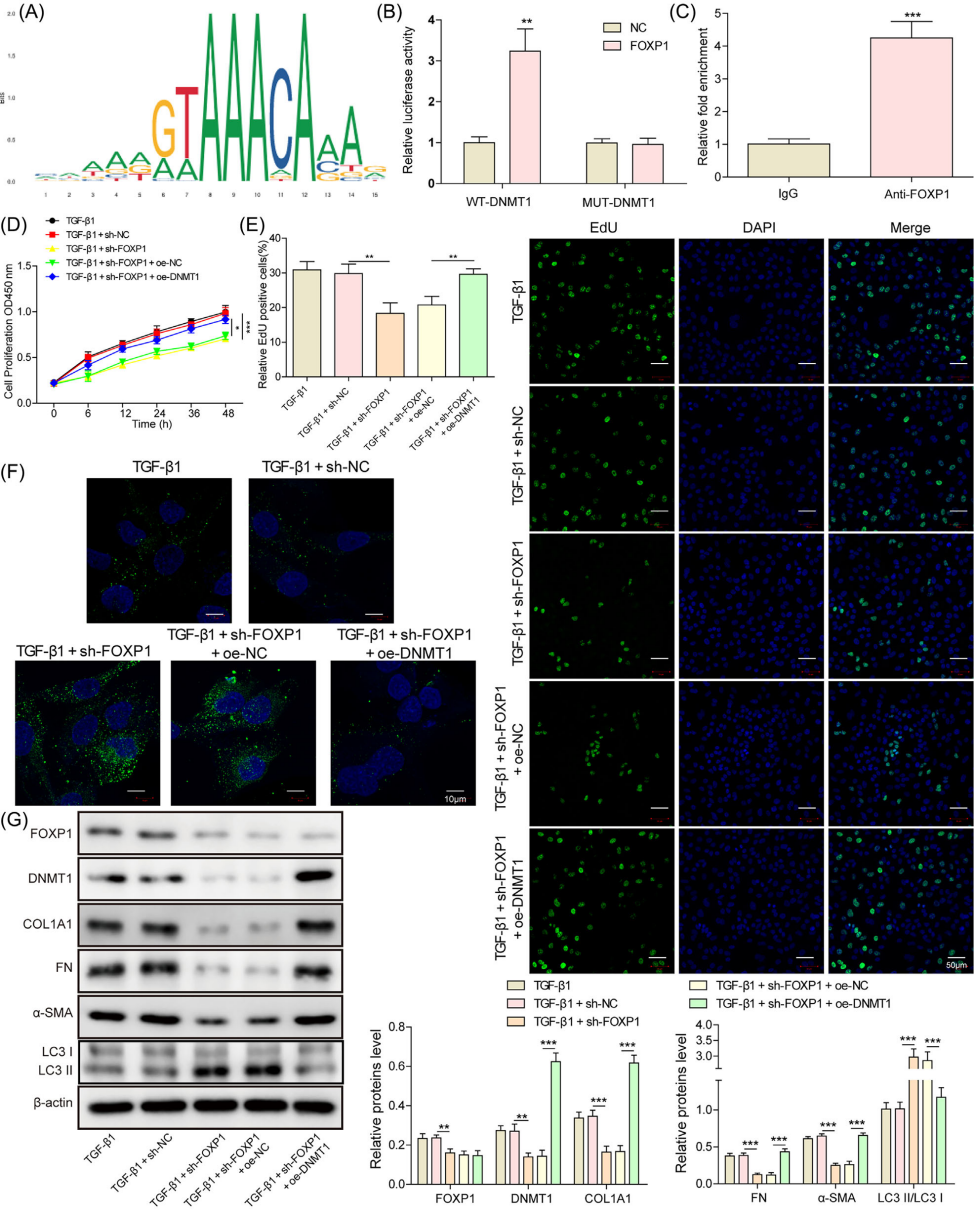
Forkhead box P1 (FOXP1) inhibits autophagy and promotes fibrosis in TGF‐β1 treated human endometrial stromal cells through transcriptionally upregulating DNA methyltransferase 1 (DNMT1). (A) The JASPAR website predicts the binding relationship of FOXP1 to DNMT1; Dual luciferase reporter gene (B) and chromatin immunoprecipitation assay (C) to verify the target binding relationship of FOXP1 with DNMT1; CCK‐8 (D) and EdU staining (E) to detect the proliferative capacity of cells in each treatment group; immunofluorescence staining to detect the expression of LC3B (F); western blot to detect the expression of FOXP1, DNMT1, COL1A1, FN, α‐SMA, and LC3‐II/LC3‐I ratio (G);   **p < 0.01, and ***p < 0.001. sh‐FOXP1, shRNAs against FOXP1; sh‐negative control, shRNAs against controlled shRNA.

Since FOXP1 is a transcription factor, we next explored whether FOXP1 has a transcriptional regulatory relationship on DNMT1. The binding sites of FOXP1 in the DNMT1 promoter region were predicted (Figure 2A). The dual luciferase reporter gene (Figure 2B) and ChIP assay (Figure 2C) together showed that FOXP1 can bind to the promoter of DNMT1. The rescue experiments were designed to examine the effects of FOXP1/DNMT1 on cell viability, autophagy, and IUA cell models. Knockdown of FOXP1 inhibited the proliferation viability of TGF‐β1‐treated hESC and promoted the expression of LC3B and autophagy (Figure 2D,E). At the same time, the expression of DNMT1 and fibrogenic factors like COL1A1, FN, and α‐SMA was inhibited, whereas the ratio of LC3‐II to LC3‐I was increased (Figure 2F). Meanwhile, overexpression of DNMT1 partially abrogated the above effects of FOXP1 on the TGF‐β1‐treated hESC. These results suggest that FOXP1 inhibits cellular autophagy and promotes fibrosis in TGF‐β1‐treated hESC by transcriptionally promoting DNMT1 expression.

### 3.3 DNMT1 inhibits autophagy and promotes TGF‐β1‐treated hESC fibrosis via promoting methylation of ULK1

**FIGURE 3:**
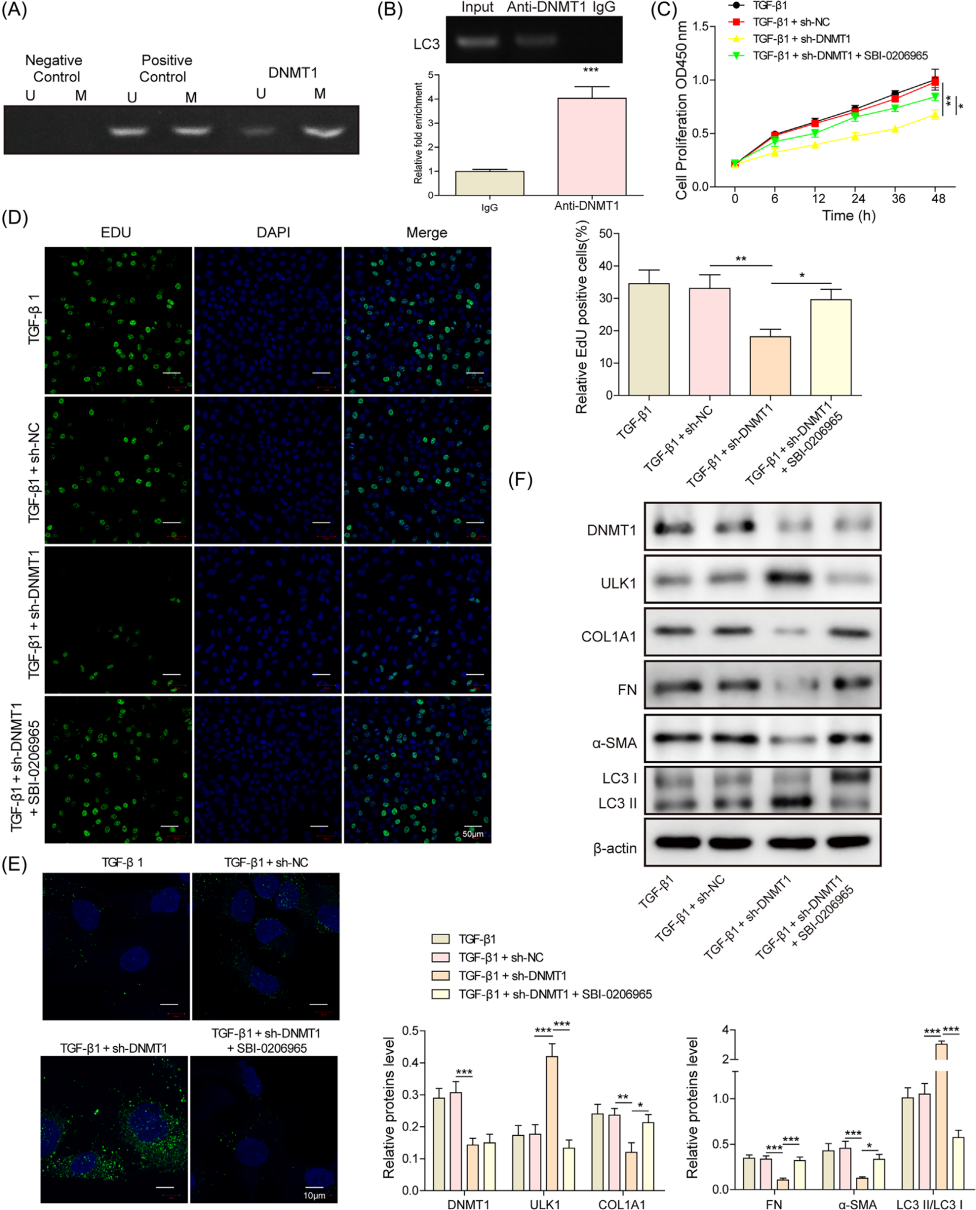
DNA methyltransferase 1 (DNMT1) inhibits autophagy and promotes TGF‐β1‐treated human endometrial stromal cells fibrosis via promoting methylation of unc‐51‐like autophagy activating kinase 1 (ULK1). (A) methylmion‐specific PCR assay to detect the methylation level of ULK1; (B) chromatin immunoprecipitation assay to detect the binding of DNMT1 and ULK1 promoter region; (C) CCK‐8 assay to detect cell proliferation; (D) EdU staining to detect cell proliferation; (E) immunofluorescence staining to detect the expression of LC3B; (F) Western blot to detect the LC3‐II/LC3‐I ratio, as well as the expression of DNMT1, ULK1, COL1A1, FN, and α‐SMA. *p < 0.05, **p < 0.01, and ***p < 0.001. sh‐DNMT1, shRNAs against DNMT1; sh‐NC, shRNAs against controlled shRNA.

The presence of CpG islands in the promoter region of ULK1 was predicted by using the online database (http://www.urogene.org/cgi-bin/methprimer/methprimer.cgi), and methylmion‐specific PCR (MSP) experiments confirmed the presence of methylation modifications in ULK1 (Figure 3A). We first investigated whether DNMT1, a classical methylation enzyme, mediates ULK1 methylation, and the ChIP experiments confirmed this hypothesis (Figure 3B). Next, the effect of DNMT1/ULK1 on cellular autophagy and fibrosis was examined. Knockdown of DNMT1 inhibited cell proliferation (Figure 3C,D), promoted the expression of LC3B (Figure 3E), and increased the protein level of ULK1 and LC3‐II/LC3‐I ratio, whereas inhibited the expression of fibrosis factors COL1A1, FN, and α‐SMA (Figure 3F). Meanwhile, SBI‐0206965 (ULK1 inhibitor) could partially reverse the effects of sh‐DNMT1. These results suggest that DNMT1 mediates the methylation of ULK1, inhibits autophagy, and promotes the fibrosis of hESC.

### 3.4 Knockdown ULK1 reverses the autophagy promotion effect of knockdown FOXP1 and aggravates TGF‐β1 treated hESC fibrosis

**FIGURE 4:**
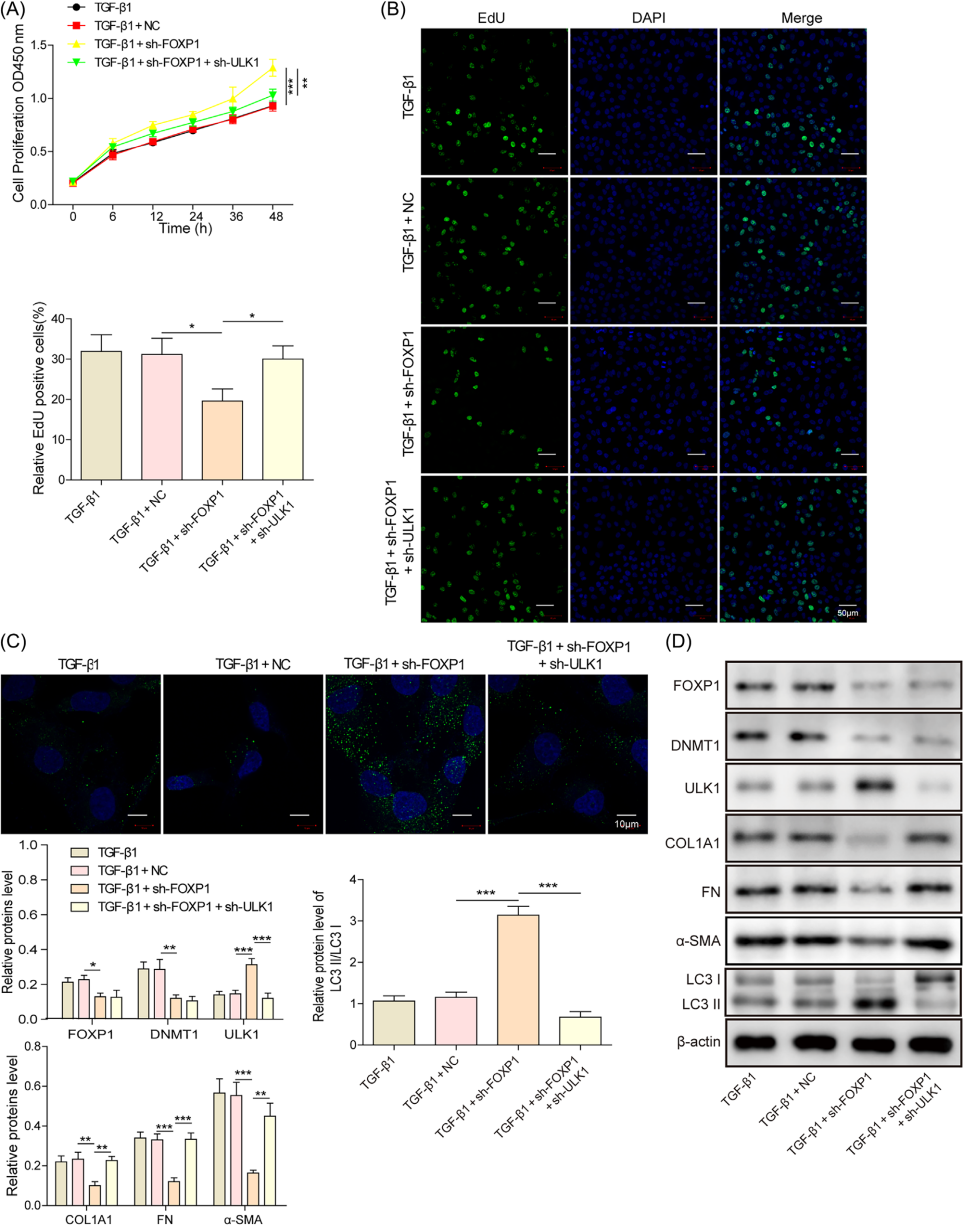
Knockdown unc‐51‐like autophagy activating kinase 1 (ULK1) reverses the autophagy promotion effect of knockdown forkhead box P1 (FOXP1) and aggravates TGF‐β1 treated human endometrial stromal cells fibrosis. (A) CCK‐8 assay to detect cell proliferation; (B) EdU staining to detect cell proliferation; (C) immunofluorescence staining to detect the expression of LC3B; (D) Western blot to detect the LC3‐II/LC3‐I ratio, as well as the expression of FOXP1, DNA methyltransferase 1 (DNMT1), ULK1, COL1A1, FN, and α‐SMA. *p < 0.05, **p < 0.01, and ***p < 0.001. sh‐FOXP1, shRNAs against FOXP1.

The previous findings that FOXP1/DNMT1 and DNMT1/ULK1 could inhibit autophagy and exacerbate hESC fibrosis, we hypothesized that FOXP1 could regulate ULK1 expression through DNMT1 and exert a regulatory effect on cell fibrosis. Consistent with the previous results, knocking down of FOXP1 inhibited cell proliferation (Figure 4A,B), promoted the expression of LC3B (Figure 4C), and increased the protein level of ULK1 and LC3‐II/LC‐I ratio, whereas suppressed the expression of DNMT1 and fibrotic factors collagen type I alpha 1 chain, FN, and α‐SMA (Figure 4D). Meanwhile, the deletion of ULK1 partially restored the above effect of FOXP1 silencing. Taken together, these results suggest that the FOXP1/DNMT1/ULK1 axis can exacerbate endometrial fibrosis by inhibiting autophagy.

### 3.5 FOXP1 inhibits autophagy to promote endometrial fibrosis in IUA rats

**FIGURE 5:**
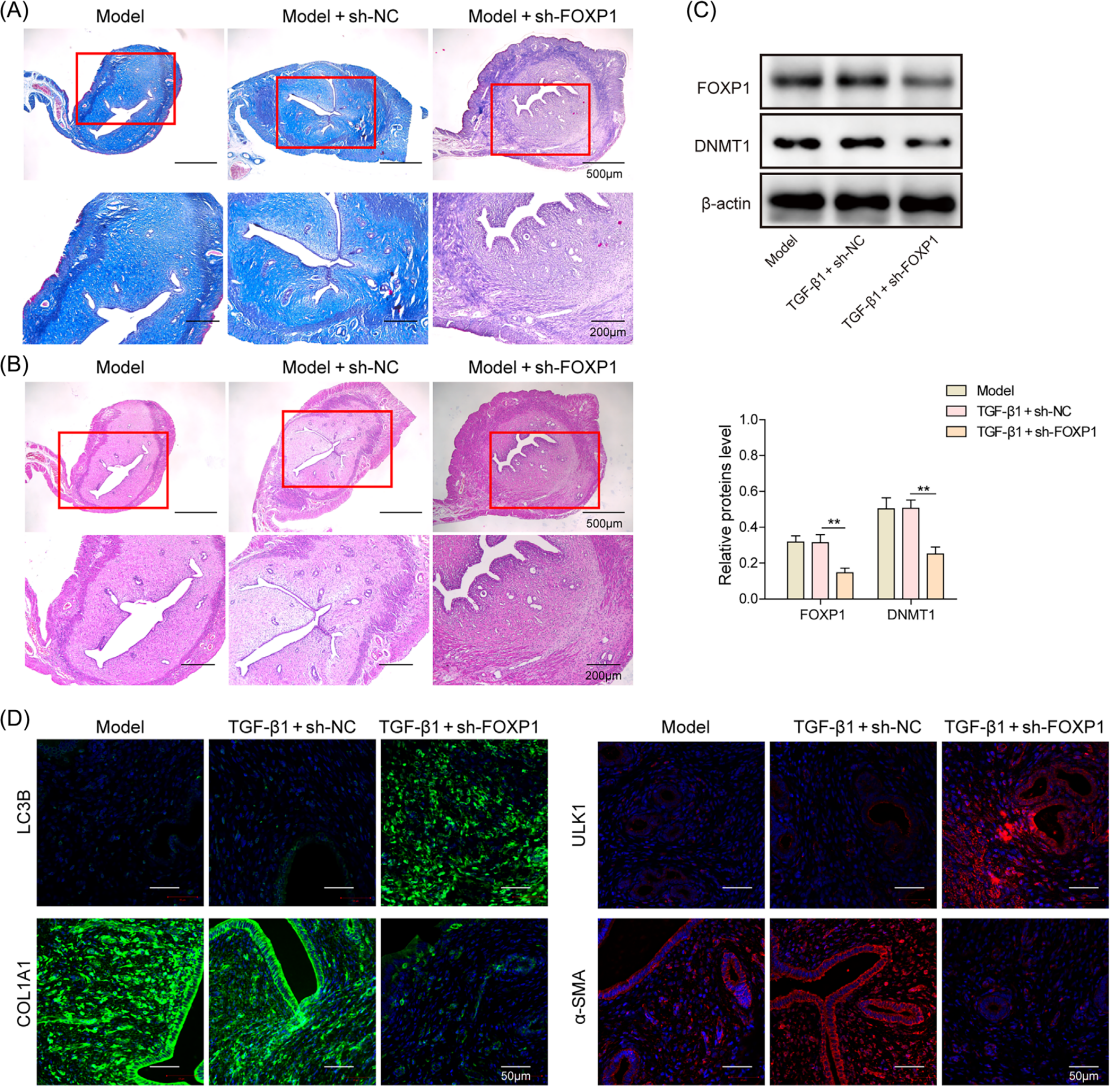
Forkhead box P1 (FOXP1) inhibits autophagy to promote endometrial fibrosis in intrauterine adhesion rats. (A) Masson staining to detect the endometrial fibrosis in rats; (B) hematoxylin and eosin staining to detect the lesions of endometrium; (C) Western blot to detect the expression levels of FOXP1 and DNA methyltransferase 1 (DNMT1); (D) immunofluorescence staining to detect the expression of unc‐51‐like autophagy activating kinase 1 (ULK1), LC3B, and fibrosis factors. **p < 0.01. sh‐FOXP1, shRNAs against FOXP1; sh‐negative control, shRNAs against controlled shRNA.

An IUA rat model was constructed, and knockdown of FOXP1 resulted in a decrease in Masson stained collagen content and a significant decrease in cellular fibrosis in uterine tissues (Figure 5A), which effectively alleviated uterine pathological adhesions (Figure 5B); in addition, knockdown of FOXP1 resulted in a significant decrease in the expression of DNMT1, fibrotic factors in uterine tissues. Meanwhile, the expression of LC3B and ULK1 was significantly elevated (Figure 5C,D). These results suggest that FOXP1 can inhibit autophagy and promote endometrial fibrosis in IUA rats by targeting and regulating DNMT1/ULK1.

## 4 DISCUSSION

This study presents novel findings regarding the functions and associated mechanisms of FOXP1, DNMT1, and ULK1 in the context of IUA. The results demonstrate that FOXP1, which is upregulated in IUA, facilitates the expression of DNMT1 through transcriptional regulation. Consequently, DNMT1 promotes DNA methylation modification of ULK1, leading to the suppression of ULK1 expression and autophagy. By inhibiting FOXP1, ULK1 expression and autophagy can be rescued, highlighting the potential of FOXP1 as a therapeutic target for the treatment of IUA.

ULK1, which is a serine/threonine kinase, plays a crucial role in autophagy regulation and is considered the most critical autophagy‐associated gene in human cells.^18^ Autophagy balance is known to have a key role in disease progression, and recent studies have observed autophagy defects in fibrotic diseases.^19^ Restoring ULK1 expression can induce protective autophagy and restore myocardial biocompetence^20^ and renal function in mice.^8^ Notably, studies have shown that autophagy is defective in the endometrium of IUA patients, which leads to increased fibrosis.^5^, ^16^ In this current study, the expression of ULK1 and autophagy marker protein LC3B decreased in both TGF‐β1‐treated hESCs and a surgery‐induced IUA animal model. These results are consistent with previous studies that suggest a possible correlation between autophagy suppression and reduced ULK1 expression in IUA.

The understanding of the role of DNA methylation in IUA is limited, making it one of the novel aspects in this study. DNA methylation plays a crucial role in the development of fibrosis in various organ systems.^21^, ^22^ Currently, DNMT inhibitors are the most widely studied and clinically used drugs for DNA methylation modification.^23^ Moreover, studies have shown that targeting DNMT1 can inhibit liver fibrosis^24^ and renal fibrosis.^25^ In the current study, we discovered a novel function of DNMT1 in IUA. It is markedly overexpressed in IUA and linked to the high expression of its upstream regulator FOXP1, which can stimulate DNMT1 transcription. Our results indicate that DNMT1 can silence the promoter region of ULK1 by facilitating its methylation.

This study reports a novel mechanism involving the interaction between FOXP1 and DNMT1. The transcription factor FOXP1 plays an important regulatory role in development, tissue homeostasis, and regeneration, as well as in several diseases. FOXP1 has been reported to promote the progression of several fibrotic diseases, respectively.^26^, ^27^, ^28^ Additionally, it has been reported that circFoxp1, derived from the Foxp1 gene, can recruit DNMT1 to the FOXP1 promoter, thereby inhibiting FOXP1 expression.^29^ In this study, we have discovered that FOXP1 can target and downregulate the expression of DNMT1, suggesting the potential existence of a feedback loop between FOXP1 and DNMT1.

Like other studies, this research has several limitations. One major limitation is the use of only hESCs in in vitro experiments, with rat models employed for further validation. The expression and function of the FOXP1/DNMT1 axis were not verified in human IUA tissue specimens. While the relationship between epigenetics, autophagy disorders, and various human diseases has been extensively studied in recent years, detecting the relevance of human autophagy in clinical trials remains challenging.^30^ This represents one of the study's limitations. Additionally, although some epigenetic drugs have demonstrated significant therapeutic effects in clinical development and several have been Food and Drug Administration‐approved, their application in disease treatment remains limited and still depends on the specific cell or disease type.^21^ Despite these limitations, the significance of this study lies in its exploration of the potential for epigenetic regulation in hESCs as a therapeutic approach for IUA.

In summary, our findings reveal that FOXP1/DNMT1 promotes ULK1 methylation modification, leading to autophagy inhibition. Furthermore, knockdown of FOXP1 or DNMT1 promoted the expression of ULK1 and LC3B. These results suggest that elevated levels of FOXP1 and DNMT1 in IUA tissues can inhibit autophagy by suppressing ULK1. Additionally, knockdown of FOXP1 partially restored the autophagy level and inhibited the expression of fibrogenic factors in IUA rats. Based on these results, FOXP1 may serve as a potential target for clinical treatment of IUA.

## CONFLICT OF INTEREST STATEMENT

The authors declare that they have no conflicts of interest.

## ETHICS STATEMENT

This study was approved by the Institutional Animal Care and Use Committee (IACUC) of Central South University on November 3, 2022 (No. CSU‐2022‐0616).

## Supporting information


**Data S1.** Supporting Information.

## Data Availability

All data generated or analyzed during this study are included in this article. The datasets used and/or analyzed during the current study are available from the corresponding author on reasonable request.
