# Supplementary material for: ULK1 methylation promotes TGF‐β1‐induced endometrial fibrosis via the FOXP1/DNMT1 axis
Source: Kaohsiung J Med Sci. 2024 Dec 4;41(1):e12915. doi: 10.1002/kjm2.12915 (PMC11724166; doi:10.1002/kjm2.12915)
Supplement: Supplementary file 1 — Data S1. Supporting Information. [file KJM2-41-e12915-s001.docx]

**Supplementary materials**

**Supplementary Table 1. Antibodies**

| **Antibody** | **Brand** | **Dilution ratio** |
| --- | --- | --- |
| EdU staining proliferation | Ab222421, Abcam | 1:1000 (western blot) |
|  |  | 1:50（IHC） |
| FOXP1 | #4402, CST | 1:1000 (western blot) |
|  |  | 1:100（IHC） |
|  |  | 1:50（ChIP） |
| DNMT1 | #5032, CST | 1:1000 (western blot) |
|  |  | 1:100（IHC） |
| ULK1 | #8054, CST | 1:1000 (western blot) |
| COL1A1 | #81375, CST | 1:1000 (western blot) |
| FN | #26836, CST | 1:1000 (western blot) |
| α-SMA | #19245, CST | 1:1000 (western blot) |
| LC3B | #83506, CST | 1:800 (Immunofluorescence) |
|  |  | 1:50（IHC） |

**Supplementary Table 2. Primer sequence**

| Name |  | Sequences (5’-3’) |
| --- | --- | --- |
| FOXP1 | Forward | CTTGCTCAAGGCATGATTCC |
|  | Reverse | CCTTGGTTCGTCAGCCAGTA |
| ULK1 | Forward | CCACCCAGTTCCAAACACCT |
|  | Reverse | CCAACTTGAGGAGATGGCGT |
| GAPDH | Forward | GGAGCGAGATCCCTCCAAAAT |
|  | Reverse | GGCTGTTGTCATACTTCTCATGG |
| DNMT1-ChIP | Forward | TCTGGATTCCAAGGCACC |
|  | Reverse | TGCGCGTGCCCTATGATG |
| ULK1-MSP-1 | Forward | GTTGTAGTGTAGTGGGGTAGTTTTC |
|  | Reverse | CTAACCAACATAATAAAACATCGTC |
| ULK1-MSP-2 | Forward | TTGTAGTGTAGTGGGGTAGTTTTC |
|  | Reverse | CTAACCAACATAATAAAACATCGTC |
| ULK1-MSP-3 | Forward | GTAGTGTAGTGGGGTAGTTTTCGT |
|  | Reverse | CTAACCAACATAATAAAACATCGTC |
| ULK1-MSP-4 | Forward | TGTAGTGTAGTGGGGTAGTTTTCG |
|  | Reverse | CCTAACCAACATAATAAAACATCGTC |
| ULK1-MSP-5 | Forward | GTTGTAGTGTAGTGGGGTAGTTTTC |
|  | Reverse | TAACCAACATAATAAAACATCGTC |
